# Targeting calcium regulators as therapy for heart failure: focus on the sarcoplasmic reticulum Ca-ATPase pump

**DOI:** 10.3389/fcvm.2023.1185261

**Published:** 2023-07-18

**Authors:** Changwon Kho

**Affiliations:** Division of Applied Medicine, School of Korean Medicine, Pusan National University, Yangsan, Republic of Korea

**Keywords:** heart failure, therapy, sarcoplasmic reticulum Ca-ATPase, calcium homeostasis, SERCA regulators

## Abstract

Impaired myocardial Ca^2+^ cycling is a critical contributor to the development of heart failure (HF), causing changes in the contractile function and structure remodeling of the heart. Within cardiomyocytes, the regulation of sarcoplasmic reticulum (SR) Ca^2+^ storage and release is largely dependent on Ca^2+^ handling proteins, such as the SR Ca^2+^ ATPase (SERCA2a) pump. During the relaxation phase of the cardiac cycle (diastole), SERCA2a plays a critical role in transporting cytosolic Ca^2+^ back to the SR, which helps to restore both cytosolic Ca^2+^ levels to their resting state and SR Ca^2+^ content for the next contraction. However, decreased SERCA2a expression and/or pump activity are key features in HF. As a result, there is a growing interest in developing therapeutic approaches to target SERCA2a. This review provides an overview of the regulatory mechanisms of the SERCA2a pump and explores potential strategies for SERCA2a-targeted therapy, which are being investigated in both preclinical and clinical studies.

## 1. Introduction

**Figure 1:**
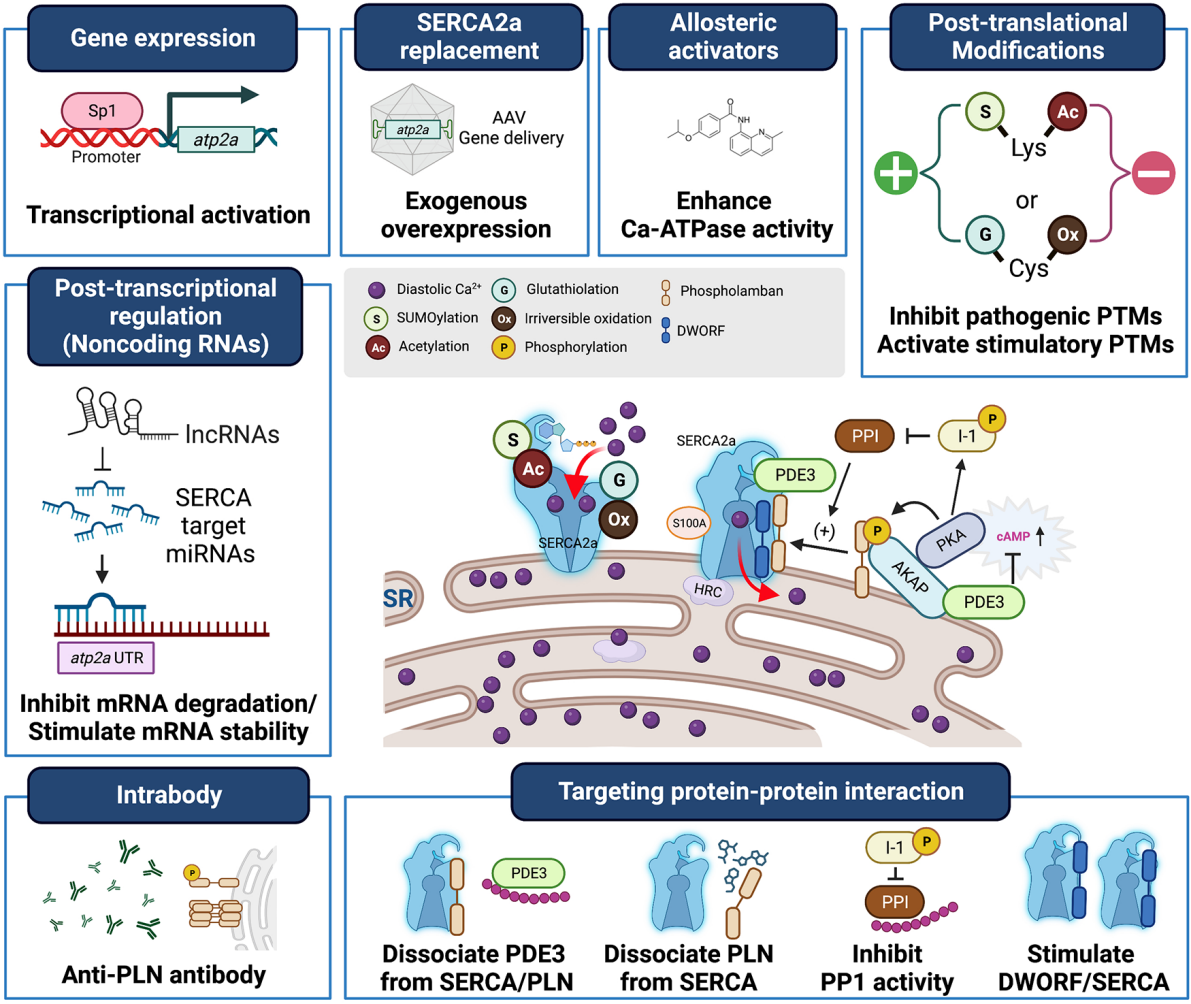
Heart failure treatment strategies targeting SERCA2a stimulation. SERCA2a deficiency is a characteristic of heart failure, and gene transfer therapies aimed at restoring SERCA2a expression are being developed clinically. In addition, new mechanisms, including post-translational modifications, non-coding RNAs, and binding partners have been identified as intrinsic modulators of SERCA2a. These will provide new targets for antibodies, peptide inhibitors and pharmacological interventions and additional opportunities to advance SERCA2a-targeted therapies.

Heart failure (HF) is a significant global health problem, affecting millions of people worldwide and becoming increasingly prevalent due to the aging population and the chronic nature of the disease (1, 2). Abnormal Ca^2+^ cycling significantly impairs cardiac contractility and repolarization in HF. Reduced total Ca^2+^ cycling, systolic free intracellular Ca^2+^ levels, and SR Ca^2+^ uptake have been demonstrated in human myocardium (3–5). Meanwhile, diastolic Ca^2+^ levels are elevated, and the duration of Ca^2+^ transients during diastole is extended compared to nonfailing hearts (4, 6). Growing evidence also indicates that changes in the expression or activity of SR Ca^2+^ cycling proteins, particularly SR Ca^2+^-ATPase (SERCA2a), are responsible for the altered Ca^2+^ cycling observed in failing hearts (7–9). This review summarizes the important role played by the SERCA2a pump in HF and the factors that regulate gene, protein, and post-translational modification (PTM) levels (Table 1). Additionally, the review discusses recent studies on how to target SERCA2a for the treatment of HF using these regulatory mechanisms (Figure 1, Table 2).

**Table 1:** Regulatory key factors of SERCA2a pump.

Category	Name	Molecular outcome	Refs
Transcription factors	Sp1	Inhibition of expression	(41)
	HIF1		(42, 43)
	NFkB		(44)
Noncoding RNAs	miR-25	Inhibition of expression	(48, 53)
	miR-132/212		(58)
	lncRNA ZFAS1		(62)
	CircITCH	Upregulation of expression	(64)
Binding proteins	PLN/I-1	Inhibition of activity	(65–67)
	DWORF	Stimulation of activity	(81)
	HRC	Inhibition of activity	(89)
	S100A1	Stimulation of activity	(90)
	PDE3A	Inhibition of activity	(97, 98)
PTMs	Oxidation	Inhibition of activity	(101, 103)
	Acetylation	Inhibition of activity	(104, 105)
	Glutathionylation	Stimulation of activity	(108, 111)
	SUMOylation	Stimulation of activity & stability	(114, 117–120)

**Table 2:** Different types of SERCA2a pump activators.

Class	Name	Mechanism of SERCA2a activation	Refs
Small molecule	CDN1163	Allosteric activation by inhibiting SERCA/PLN	(125–128)
Small molecule	Compound A	Allosteric inhibition of PLN	(129)
Small molecule	Istaroxime	Allosteric inhibition of PLN & SERCA/PLN	(130, 131)
Small molecule	PST3093	Istaroxime metabolites	(136, 137)
Antibodies	PLN intrabody	Allosteric inhibition of PLN	(140)
Peptide	PP1 decoy	Inhibition of dephosphorylation of PLN	(76)
Peptide	DWORF	Allosteric activation	(81–87)
Peptide	OptF	Inhibition of PDE3A/SERCA2a	(141)
Small molecule	N106	Activation of SUMOylation	(120)
Small molecule	Ginsenoside Rg3	Activation of SUMOylation	(142)
Small molecule	Luteolin	Activation of SUMOylation, Sp1, phospho-PLN	(144, 145)
Small molecule	β-lapachone	Activation of deacetylation	(104)
Small molecule	Resveratrol	Enhancement of transcription	(147, 148)

## 2. SERCA2a: a key calcium-handling protein

Excitation–contraction coupling (ECC) is a well-established process that regulates the fundamental functions of cardiomyocytes (CMs) such as contraction and relaxation (10). In response to electrical depolarization, the opening of the L-type Ca^2+^ channel (LTCC) leads to Ca^2+^ influx and subsequent release of Ca^2+^ from the SR via ryanodine receptors (RyRs). The resulting increase in cytosolic Ca^2+^ triggers muscle contraction through the binding of Ca^2+^ to myofibrillar protein troponin C, while relaxation occurs when cytosolic Ca^2+^ is isolated back into the SR by SERCA2a and is released by other mechanisms such as Na^+^/Ca^2+^ exchange (NCX), sarcolemma Ca^2+^-ATPase, mitochondrial Ca^2+^ uniporter. Given this key role of ECC in regulating CM function, abnormalities in SR Ca^2+^ homeostasis in ventricular CMs are expected to significantly impact cardiac performance.

### 2.1. Pathophysiological role of SERCA2a in the heart

SERCA2a is an SR membrane protein that maintains low cytosolic Ca^2+^ levels by carrying out Ca^2+^ transport from the cytosol into the SR. The SERCA2a pump is responsible for approximately 70% and 92% of Ca^2+^ ventricular CM turnover during the diastolic period in humans and rats, respectively (11, 12). At this time, the Ca^2+^ removal rates through NCX are 28% and 7%, respectively, and the remaining approximately 1% of active Ca^2+^ is removed by the sarcolemmal Ca^2+^ ATPase and mitochondrial Ca^2+^ uniporter (11, 12).

The physiological consequences of SERCA2a disruption were studied in heterozygous knockout (KO) mouse models (13, 14). SERCA2a KO mice showed a decreased rate of SR Ca^2+^ sequestration and deficiencies in the relaxation and contraction of CMs as a direct result of a reduction in SERCA2a levels. Biochemical studies confirmed a decrease in mRNA levels (∼55%), protein expression, and activity (∼65%) of the SERCA2a pump in the hearts of heterozygote KO mice (13). Additionally, a study involving a cardiac-specific conditional KO mouse model demonstrated the physiological importance of SERCA2a for cardiac performance in the adult heart. CM-specific SERCA2a-deficient mice exhibited severe diastolic dysfunction *in vivo* (15). Of note, the CM-specific SERCA2a KO develops end-stage HF within 7 weeks (15). It is interesting that both the SERCA2a KO and the NCX KO mice, another major Ca^2+^ handling protein, are able to robustly compensate and maintain normal cardiac function over a long period of time (16). Despite little change in cardiac function *in vivo* at 4 weeks after gene ablation, reduced contraction observed in SERCA2a-deficient CMs suggests that there may be a mouse-specific factor that allows them to maintain function despite the loss of key proteins. The faster heart rate seen in mice compared to humans may act as a compensatory factor by promoting efficient physiological function by promoting rapid delivery of oxygen and nutrients to tissues.

Studies have reported decreased SERCA2a expression and/or activity in the hearts of patients with dilated cardiomyopathy and ischemic cardiomyopathy (7–9, 17). Furthermore, a critical correlation has been observed between SERCA2a protein levels and cardiac function, as assessed by a force-frequency relationship (18). Excessive diastolic Ca^2+^ accumulation can lead to prolonged relaxation times, which in turn can increase chamber stiffness and decrease the rate of systolic pressure generation. Furthermore, reduced SERCA2a activity has been linked to arrhythmogenicity, as it can lead to diastolic Ca^2+^ accumulation and spontaneous Ca^2+^ releases from the SR, which are considered one of the cellular substrate for arrhythmia (19). Despite reduced SR Ca^2+^ stores, failing CMs show enhanced spontaneous SR Ca^2+^ release events (20), which may involve a reduced threshold for SR Ca^2+^ leak as observed in SERCA2a inhibition studies (21). Recent studies have shown that SERCA2a activation reduces the occurrence of spontaneous diastolic SR Ca^2+^ leak (22). Treatment with small molecule stimulators of SERCA2a was found to increase the threshold for SR Ca^2+^ wave generation and slow Ca^2+^ wave propagation (22). On the other hand, phospholamban (PLN), an inhibitory protein of SERCA2a, can cause severe arrhythmic cardiomyopathy with certain mutations, such as the deletion of arginine 14, which is particularly prevalent in the Dutch population (23). The SERCA2a super-inhibitory phenotype of this variant has been reported (24), supporting an aberrant SERCA2a role in arrhythmias.

Enhancing or restoring SERCA2a activity has been shown in multiple studies to improve cardiac function and prevent the progression of HF in various animal models, providing strong evidence for the causal role of SERCA2a downregulation in HF progression.

Studies on transgenic mice demonstrated that re-induced SERCA2a was well expressed in adult hearts and that increased expression of the SERCA2a pump directly enhances cardiac contractility in HF (25). Numerous preclinical studies on rodents, pigs, and sheep as well as on CMs from human patients with HF, have demonstrated the feasibility of restoring SERCA2a function through gene therapy in various HF models including acute ischemia/reperfusion, chronic pressure overload, and chronic myocardial infarction (MI) with particular focus on HF with reduced ejection fraction (HFrEF) (25–28). Unlike positive inotropes, which can be arrhythmogenic and associated with sudden death, restoration of SERCA2a expression has been shown to prevent ventricular arrhythmias and improves cardiac energy metabolism, suggesting clinical benefits (29–32).

Moreover, decreased SERCA2a expression has been observed in heart transplant recipients with preserved systolic ejection fraction (33), and dysfunction of SERCA2a activity has been linked to diastolic dysfunction and HF with preserved ejection fraction (HFpEF) in the senescent, obsess, and diabetic heart (34–47). To address these issues, SERCA2a gene therapy (35) and pharmacological agents such as istaroxime (38) have been investigated as potential therapeutic options to upregulate SERCA2a activity and improve diastolic function and contractile reserve in aging and HF.

## 3. Regulators at expression level

### 3.1. Transcriptional factors

Changes in gene transcription may be associated with reduced SERCA2a mRNA levels in cardiac hypertrophy and HF. Through promoter analysis, cardiac muscle-specific regulatory regions in *atp2a2* (SERCA2a gene) were identified that contain binding sites of important transcription factors responsible for the cardiac pathological environment such as specificity protein 1 (Sp1), GATA binding protein 4, serum response factor, nuclear factor of activated T-cells, and myocyte enhancer factor-2 (39, 40).

Sp1 is an essential factor for myocardial hypertrophy (HT) along with an upregulated gene, such as *acta1* (alpha-skeletal actin), in enlarged hearts. The mRNA level of Sp1 and its promoter binding activity is increased in pressure-overloaded hearts. Two Sp1 binding sites near the SERCA2 gene promoter may cause transcriptional inhibition mediated by Sp1 during cardiac HT induced by pressure overload (41).

Hypoxia-inducible factor-1 and nuclear factor kappa-B (NF-κB) are candidate transcription factors associated with pathogenic condition-induced *atp2a2* suppression. SERCA2 expression is sensitive to oxygen levels in cultured CMs (42, 43). Rat and mouse *atp2a2* gene promoters contain two hypoxia response element areas (−777 bp to −781 bp and −1,073 bp to −1,077 bp from the transcription start site), and both sites seem to be essential for repressing *atp2a2* expression. Another study reported that SERCA2a expression is downregulated by tumor necrosis factor-α (TNF-α) and NF-κB-induced mechanisms (44). Studies showed that treatment of isolated rat CMs with TNF-α induced nuclear translocalization of NF-κB through activation of IκB kinase and that NF-κB binds directly to the promoter of human *atp2a2* to inhibit transcription.

Other reports demonstrated epigenetic repression of the SERCA2a gene in HT myocardium. DNA modification and PTM of histones are the main mechanisms of epigenetic processes (45, 46). DNA methylation in the *atp2a2* promoter (approximately 3,500 bp upstream of the *atp2a2* transcriptional start site) is upregulated when pathogenic HT situations are induced by afterload enhancement (AE) in engineered heart tissue models consisting of neonatal rat CMs. Meanwhile, AE-induced *atp2a2* methylation is downregulated by pharmacological inhibitors of DNA methyltransferase (DNMT) such as RG108 (47). However, in-depth investigations are needed to clarify the role of DNMTs in cardiac HT and HF.

Recently, evidence suggests that epigenetic mechanisms can control microRNA (miRNA) expression. Studies have shown that expression of miR-25, a SERCA2a target miRNA, is positively regulated by the transcriptional co-factor sine oculis homeobox homolog 1 (Six1). Abnormal hypermethylation of CpG islands adjacent to the Six1 promoter was observed in failing hearts, suggesting a decrease in the epigenetic inhibition of Six1 (48).

### 3.2. microRNAs

Small (<200 nucleotides) non-coding RNAs, including microRNAs (miRNAs), have emerged as crucial gene regulators, and extensive functional studies have demonstrated their significant involvement in key pathogenic mechanisms of HF, including HT and arrhythmia (49–51). Several miRNAs that directly target *atp2a2* have also been reported.

MiR-25 has been investigated for its *in vivo* role in Ca^2+^ handling and cardiac remodeling. MiR-25 belongs to the miR-106b-25 cluster and resides in the intron of the minichromosome maintenance complex component 7 gene (52). In pressure-overloaded mice, overexpression of miR-25 induced cardiac HT and HF (53). Meanwhile, inhibition of miR-25 by injection of specific antisense oligonucleotides (antagomir) (53) or adeno-associated virus (AAV) vectors encoding miR-25 tough decoy (54) improved cardiac contractility and increased SERCA2a expression. In fact, miR-25 expression was elevated in failing human myocardia (53); however, another mouse model study reported the opposite effect of miR-25 (55). In that study, miR-25 expression in the heart was downregulated in pressure overload. Antagomir-25 treatment accelerated pressure overload-induced cardiac dysfunction and fibrosis. Furthermore, the proposed mechanism of action on miR-25 was interpreted as a result of overactivation of the heart and neural crest derivatives that expressed 2 transcription factors. This discrepancy between cardiac phenotypes can be caused by differences in experimental design, including the disease severity and dose, and duration of antagomir treatment (56). Additionally, there are several candidate miRNAs that directly target SERCA2a, including miR-29c (43), miR-328 (57), and miR-132/212 (58). Importantly, a phase 2 clinical trial (ClinicalTrials.gov, Identifier: NCT05350969) is currently underway to evaluate the effectiveness and safety of an antisense inhibitor of miR-132a in a patient with HF following MI. A small cohort phase 1b study involving CDR132l, an inhibitor of miR-132 that reduces SERCA2 expression, showed promising results in terms of safety and effectiveness, highlighting the potential of miRNA inhibitors in the treatment of HF (59).

### 3.3. Long non-coding RNAs

Long non-coding RNAs (lncRNAs) are >200 nucleotides long and function as signals or guides, scaffolds, or decoy molecules, and the cell, tissue, or disease-specific expression patterns of lncRNAs make them appealing as potential diagnostic and treatment targets for complex diseases, such as heart disease (60). Located in the antisense strand adjacent to the 5′ end of the Zinc Finger NFX1-Type Containing 1 coding gene (*Znfx1*), *Znfx1* antisense RNA (ZFAS1) (61) has been proposed as an oncogenic lncRNA in almost all types of human malignant tumors. Interestingly, ZFAS1 expression is upregulated in ischemic human and mouse heart tissues (62). Studies on the regulation of *in vivo* ZFAS1 expression through the AAV system have revealed that ZFAS1 overexpression negatively affects cardiac contractility in MI mice models (62). These physiological results were due to the ability of the ZFAS1 molecule to bind and directly inhibit SERCA2a. However, further experimental evidence is needed to second the regulatory mechanism of ZFAS1 on SERCA2a.

### 3.4. Circular RNAs

Circular RNAs (circRNAs) ranging in size from hundreds to thousands of nucleotides, with their covalent closed-loop structure and ability to function as miRNA and RNA-binding protein sponges, are gaining attention as a novel modality for cancer treatment (63). Specific circRNAs involved in tumor development are associated with cardiac pathologies. CircRNA produced from the E3 ubiquitin-protein ligase *ITCH* gene (circITCH) with antitumor activity has been reported in CMs (64). CircITCH expression was reduced in response to doxorubicin treatment both in patients and human-induced pluripotent stem cell-derived CMs models. In a mouse study, AAV-mediated circITCH overexpression prevented doxorubicin-induced cardiac dysfunction and structural remodeling (64). As a mechanism of action, it was proposed that circITCH functions as a competitor of miR-330-5p, which targets the SERCA2a 3′-untranslated region (64).

## 4. Peptide regulators of SERCA2 activity

### 4.1. Phospholamban (PLN)

PLN, a phosphoprotein predominantly expressed in the heart, interacts with SERCA2a in its dephosphorylated state, causing conformational changes that reduce the pump's affinity for Ca^2+^ and slow Ca^2+^ uptake into the SR (65, 66). Both protein kinase A (PKA) and Ca^2+^/calmodulin-dependent protein kinase II (CaMKII) induce cAMP-dependent phosphorylation of PLN, alleviating its inhibitory effect on SERCA2a (66). SR-related protein phosphatases, including type I protein phosphatase (PP1), contribute to the reversible phosphorylation of PLN (67). A kinase anchored protein 18delta (AKAP18δ), an adaptor protein, plays a crucial role in recruiting PKA and CaMKII to the SR membrane, facilitating PLN phosphorylation in response to adrenergic stimuli (68, 69). Disruption of PLN–AKAP binding leads to reduced PKA-dependent PLN phosphorylation and impaired SERCA2a activity (68). Additionally, AKAP18δ can form a complex with CaMKIIδ, with binding at different regions regulating its activity in an inverse manner (69). Frequency-dependent double phosphorylation induced by AKAP18δ–CaMKII has been proposed as an adaptive mechanism for the Ca^2+^ pacing frequency in CMs. Moreover, AKAP18 can modulate PP1 activity by interacting with inhibitor (I-1), a cAMP-regulated phosphoprotein that suppresses PP1 (70). These complex regulatory mechanisms involving PLN phosphorylation and its interaction with various signaling molecules will contribute to the fine-tuned control of intracellular Ca^2+^ handling in the CM.

In failing human hearts, PLN levels remain stable, whereas SERCA2a levels are decreased, resulting in an increase in the PLN/SERCA2a ratio. AAV-mediated PLN antisense RNA expression enhances SERCA2a function and CM Ca^2+^ cycling (71). Meanwhile, reduced PLN expression improves cardiac contractility and HF characteristics in cardiomyopathic mice (72). Additionally, PLN phosphorylation is decreased in failing human hearts (73, 74), which is associated with enhanced activity of PP1 (74, 75). Competitive decoys for PP1 resulted in increased phosphorylation of PLN and contractility in CMs and isolated rat hearts (76). Furthermore, PP1 inhibition through overexpression of the constitutively active form of inhibitor 1 (I-1c) rescued cardiac abnormalities in HF mice models (77). More importantly, AAV-mediated expression of I-1c was shown to be effective and did not cause serious toxicity in preclinical HF pig models (78), suggesting its potential for clinical use.

In addition to the arrhythmia-associated R14del mentioned above, two other PLN mutations, Arg9Cys and Leu39Stop, have been reported in familial dilated cardiomyopathy (79, 80). These mutations were associated with either sustained dephosphorylation or absence of PLN; regardless, both resulted in HF phenotypes. These studies suggest the significance of PLN in maintaining heart function in humans. More research on the characteristics of the different types of human PLN mutations is needed to understand their effects on the etiology of heart diseases.

### 4.2. Dwarf open reading frame (DWORF)

Misannotated several lncRNAs have been identified, and among them is DWORF, a lncRNA that encodes a muscle-specific 34-amino acid peptide serving as an activator of SERCA2a (81). In an initial study, DWORF was proposed to act as a non-inhibitory competitor for PLN that increases the activity of SERCA2a by replacing PLN bound to SERCA2a. It was suggested that Ca^2+^ dependence is the key factor for PLN/DWORF micropeptide-based SERCA2a regulation (81, 82). However, recent studies showed that DWORF could directly interact with SERCA2a without PLN (83, 84). DWORF is likely to play a role in the reciprocal regulation of SERCA2a with PLN by enhancing the binding properties of SERCA2a during a distinct enzymatic cycle, separate from that of PLN. Specifically, DWORF binds to the E1P and E2P reactive states of SERCA2a, whereas PLN binds to the E1-ATP state (84). Point mutation analysis confirmed that two residues of DWORF, namely Pro15, and Trp22, are critical for its interaction with the SERCA protein. Overexpression of DWORF enhances SERCA2a function and contractility in a DCM mouse model that genetically deleted the muscle-specific LIM domain protein (85). Additionally, decreased DWORF expression was observed in the heart of an MI mouse model, and overexpression of DWORF through the AAV9 system improved heart function following MI (86). Recently, the relevance of DWORF overexpression on cardiac pathogenesis was also reported in a mouse model of Duchenne muscular dystrophy (DMD) (87). As observed in an MI mouse heart, DWORF was reduced in the dystrophin-deficient hearts, wherein SERCA2a function was impaired (87). Enzymatic activity of SERCA, hemodynamic parameters, including maximum left ventricular pressure and ejection fraction, and histological features of cardiac fibrosis were significantly improved in hearts with DWORF overexpression (87). However, there was no improvement in the tau value, the most established index reflecting the diastolic relaxation rate of the heart (88), which is in part due to SERCA2a activity. Given its potential as a therapeutic agent, it is necessary to clarify the detailed regulatory mechanisms of DWORF-mediated SERCA2a activation in pathological settings.

## 5. Other proteins

Histidine-rich calcium-binding protein (HRC) and small Ca^2+^ binding protein A1 (S100A1) are intracellular Ca^2+^ handling proteins that interact with SERCA2a in mouse and human hearts, with their binding being Ca^2+^-dependent and involving other Ca^2+^ regulatory proteins such as RyR2 and PLN (89, 90). Specific single-nucleotide polymorphisms in HRC, such as Ser96Ala (S96A), have been associated with arrhythmogenesis in Greek patients with idiopathic dilated cardiomyopathy (91). Gene transfer of S100A1 has shown beneficial effects in preclinical models of HF, including pigs and human CMs highlighting its potential as a therapeutic target (92, 93). However, the challenge of achieving sufficient S100A1 gene delivery within safe doses limits its clinical utility in HF. Recent research has also explored using S100A1 as a diagnostic marker for HF (94).

The PDE3 inhibitor milrinone (Primacor) is used in the treatment of acute decompensated HF, but its long-term use is limited due to the risk of ventricular tachycardia and increased mortality (95). Studies using subtype-specific knockout mice have provided evidence for the role of PDE3A in regulating cardiac inotropic function (96), with PDE3A forming protein complexes in the SR membranes that include AKAP18δ, PKA, PLN, and SERCA2a. Phosphorylated PDE3A recruited to these complexes modulates SERCA2a activity through cAMP-dependent mechanisms by reducing cAMP levels, inhibiting PKA activation, and decreasing PLN phosphorylation, ultimately leading to a reduction in SERCA2a activity (97, 98). Furthermore, PDE3A directly interacts with SERCA2a in a phosphorylation-dependent manner, suggesting the potential for targeting PDE3A by interfering with the interaction between these two proteins (98).

## 6. Post-translational modifications

### 6.1. Oxidation

Increased reactive oxygen species (ROS) and oxidative stress are clinically involved in the onset and progression of HF, leading to cellular damage and disease pathogenesis (99). Notably, ROS modification can directly impact cardiac contraction. For instance, the irreversible sulfonylation of SERCA at Cys674 has been demonstrated to disrupt Ca^2+^ dysregulation and impair contractile function in failing (100) and senescent mouse hearts (101). Another oxidative modification of interest is tyrosine nitration, which involves the addition of a nitro group (-NO2) to position 3 of the aromatic ring of tyrosine residues. These modifications can induce significant structural and functional changes in proteins due to alternations in biochemical properties such as local pKa, redox potential, hydrophobicity, and volume (102). Tyrosine-nitrated proteins have been detected in various pathological conditions and are also associated with aging. In failing and senescent hearts, nitration of Tyr294/Tyr295 of SERCA has been observed (100, 103). This nitration event, occurring near the Ca^2+^ translocation region in the transmembrane site, is thought to disrupt the helix–helix interaction and interferes with the membrane action required for optimal SERCA activity rates. However, the precise physiological implications of nitrotyrosine modification have yet to be fully elucidated.

### 6.2. Acetylation/deacetylation

Protein acetylation of lysine residues a reversible and one of the most common types of PTM, and studies have demonstrated the significance of SERCA2a acetylation in HF. Acetylation of Lys492 in SERCA2a reduces Ca-ATPase activity and CM contractility, and elevated levels of acetylated SERCA2a have been observed in HF patients and pressure overload-induced HF models (104). Animal studies employing gain-of-function and loss-of-function approaches have provided evidence that the acetylation status of Lys492 in SERCA2a is regulated by the deacetylase activity of sirtuin 1 (SIRT1). Moreover, treatment of mice with transverse aortic constriction with a SIRT1 activator, such as β-lapachone, demonstrated a preventive effect on pathological structural remodeling. This treatment also reduced SERCA2a acetylation and increased its activity, leading to improved cardiac contractility (104). Another study provided an additional regulatory mechanism of SERCA2a acetylation focused on p300 acetyltransferase, which is required for SERCA2a acetylation (105). In that study, 12 putative acetylated lysines, including Lys492 in SERCA2a mediated by the p300 enzyme, were identified by mass spectrometry analysis. As a result of analyzing biochemical characteristics using an acetylation mimic mutant substituted lysine residue with glutamine, K514Q showed reduced Ca-ATPase activity compared to wild-type and un-acetylated mutant K514R. Moreover, K514Q showed less protein stability than K492Q SERCA2a. The K514Q knock-in CMs also showed impaired contractile properties similar to those of K492Q. These results suggest that the modification of important residues for proper ATP binding, such as Lys492 and Lys514, are highly sensitive to the function of SERCA2 (106). Each acetylated lysine can have diverse effects on various aspects of SERCA2a function, underscoring the need for further in-depth research. Notably, acetylation of non-histone proteins is a critical regulatory mechanism of protein–protein interactions (107). In this regard, the study proposed the involvement of acetylation in the SERCA2a–PLN complex by identifying acetylation in Lys368, an essential residue for binding to PLN. Interestingly, the K368Q mutant of SERCA2a showed increased binding affinity to PLN compared to the wild-type or other KQ mutants, suggesting the potential role of acetylation in modulating the SERCA2a–PLN interaction.

### 6.3. Glutathiolation

*S*-glutathiolation is a reversible cysteine modification that results from a reaction between glutathione (GSH, oxidized form) with free thiol. Among the glutathiolated cysteines, Cys674 in SERCA2a is considered the most reactive thiol (108). The oxidation of SERCA2a Cys674 can either stimulate (via reversible glutathiolation) or inhibit (via irreversible sulfonylation) Ca-ATPase activity. In vascular smooth muscle cells, nitric oxide (NO) induces SERCA glutathiolation and enhances SERCA Ca-ATPase activity (109). Nitroxyl (HNO), the one-electron reduced product of NO, exhibits positive inotropic and lusitropic effects on the heart (110). HNO induces biochemical changes in the SERCA2a pump and RyR2 channels in CMs, increasing Ca^2+^ uptake and release from the SR without triggering abnormal Ca^2+^ homeostasis (111). In that study, treatment of HNO donor Angeli's salt (AS) increased SERCA2a glutathiolation and SERCA2a activity by approximately 20% and 40%, respectively, in isolated adult rat CMs. In cells overexpressed with wild-type SERCA2b, AS increased SERCA activity by approximately 60%. However, this stimulating effect of AS was not observed in cells overexpressing the C674S mutation of SERCA2b. In a mouse model with the C674S point mutation, which replaced 50% of SERCA Cys674 with serine, CM contractility was reduced (112). The delayed time constant of the intracellular Ca^2+^ decay (tau values) in C674S mutant mice suggested reduced SERCA activity (112). These findings highlight the significance of cysteine oxidation in both the physiological and pathological aspects of SERCA function. Other studies have shown that PLN contributes to SERCA2 activation through modification of HNO-induced thiol residues or stability of oligomers (113). However, additional research is required to understand the mechanisms of SERCA in glutathiolation and identify the endogenous sources of HNO, particularly *in vivo*.

### 6.4. SUMOylation

SUMOylation is a reversible modification wherein a small ubiquitin-like modifier (SUMO) protein conjugates to a lysine residue in a substrate protein, and aberrant SUMOylation has been linked to HF etiology (114–116). Biochemical analysis combined with point mutation studies demonstrated that SERCA2a is the substrate of SUMO1 and that SUMOylation on Lys480 and Lys585 increases Ca-ATPase activity and stabilizes SERCA2a (114). Levels of SERCA2a SUMOylation and SUMO1 were significantly reduced in both human and animal HF (114). Mice that knocked down cardiac SUMO1 expression via AAV9-SUMO1-short hairpin RNA injection accelerated contractile dysfunction and pathological remodeling (114). Meanwhile, restoring SUMO1 expression through gene manipulation (e.g., heart-specific SUMO1-transgenic mice) or gene therapy (e.g., AAV9 vector encoding SUMO1) in mice with HF improved cardiac performance and enhanced the survival rate (114). The therapeutic efficacy of AAV-mediated SUMO1 gene transfer was further confirmed in MI pig models (117). Under pathogenic conditions, SUMO1 restoration improved cardiac function by increasing SERCA activity through improved SERCA2a SUMOylation along, as well as exerted its antioxidant effects (118). Interestingly, SUMO1 restoration significantly reduces SERCA2a oxidation induced by tyrosine nitration, suggesting the protective role of SUMOylation on SERCA2a under pathogenic conditions. Subsequent studies further confirmed that abnormal elevation of miR-146a is involved in SUMO1 deficiency in failing hearts (119). MiR-146a is found in exosomes secreted from myofibroblast and transfers into CMs. Furthermore, a small molecule activator of SERCA2a SUMOylation, namely N106, was discovered (120), which confirmed the efficacy in mice with HF. These results indicate the pathophysiological significance of SERCA2a SUMOylation and its potential as a therapeutic target. In this regard, further in-depth understanding of the therapeutic ranges of SUMOylation and cross-talk with other PTMs is needed.

## 7. Therapeutic approaches targeting SERCA2a

### 7.1. Clinical development of SERCA2a gene therapy

AAVs vectors have been developed for delivery in cardiac clinical trials, including studies such as CUPID 1/2, AGENT-HF, and SERCA-LVAD, to evaluate the safety, feasibility, and efficacy of AAV-mediated SERCA2a gene therapy (121–123). The CUPID trials demonstrated the safety and tolerability of AAV1.SERCA2a in patients with advanced HF (NYHA class III/IV). The CUPID phase 1 and early phase 2 trials demonstrated the safety and potential efficacy of intracoronary infusion of AAV1.SERCA2a in improving HF symptoms, left ventricular structure and function, and increasing time to cardiac events. However, the subsequent CUPID-2 trial did not meet its primary endpoints, leading to the premature termination of the AGENT-HF and SERCA-LVAD trials. The exact reason for the failure of the CUPID-2 trial is not fully understood, but the inadequate dosage was identified as a contributing factor to the neutral results of the study (121). As a result, the ongoing CUPID-3 trial (NCT04703842) for patients with HFrEF has implemented an increased therapeutic dose of AAV1.SERCA2a (SRD-001). In December 2022, the U.S. Food and Drug Administration approved an investigational new drug application for SRD-001 in treating cardiomyopathy associated with DMD, making it the first-in-human gene therapy for DMD cardiomyopathy, a leading cause of death in end-stage DMD patients.

### 7.2. Clinical development of I1-c gene therapy

A recent phase 1 clinical trial (NCT04179643) has been initiated to investigate the potential of I-1c gene therapy in treating patients with NYHA class III HF. The therapy aims to enhance cardiac function by increasing the phosphorylated form of PLN, which is achieved through the inhibition of PP1. The study utilizes an AAV.I-1c vector for delivery, with the BNP116 vector (also called AAV2i8) being the newly designed delivery system. The BNP116 vector incorporates CM-specific receptor binding regions, enhancing cardiac tissue selectivity and efficiency. This novel vector design may be more suitable for translational studies involving cardiac gene delivery compared to conventional AAV vectors (124).

### 7.3. SERCA allosteric activators

Small molecule CDN1163 (4-[1-Methylethoxy)-N-[2-methyl-8-quinolinyl]-benzamide) was discovered as a pan-SERCA allosteric activator through SERCA–PLN interaction via fluorescence resonance energy transfer and sequential medicinal chemical optimization (125). Studies on *in vitro* absorption, distribution, metabolism, excretion (ADME) analysis, and mouse pharmacokinetics (PK) provided an initial understanding of the pharmacological properties of CDN1163 (126). The efficacy of the CDN1163 has been studied in diverse animal models of human diseases, including diabetes, Parkinson's disease, and DMD (126–128). Recently, the beneficial effects of CDN1163 have been reported in dystrophin-deficient MDX mice (128). These mice were intraperitoneally injected with 40 mg/kg of CDN1163 for 7 weeks, resulting in improvements in skeletal muscle degeneration and fibrosis compared to the DMSO injection group. Additionally, the grip strength of the MDX mice was improved with CDN1163 treatment. However, CDN1163 treatment did not have an effect on muscle hypertrophy, which is considered a compensatory mechanism of muscle degeneration. It is important to note that dose-dependent experiments with CDN1163 have not been performed extensively due to the compound's low solubility. While CDN1163 shows potential, its effects on the heart were not evaluated in this particular study.

### 7.4. PLN–SERCA2a dissociation

Compound A is a novel pyridone derivative with SERCA2a inhibitory activity that targets PLN (129). Surface plasmon resonance experiments have demonstrated the direct binding of compound A to PLN within the μM range (129). In that study, physiological effects of Compound A were investigated in both isolated normal rat hearts and normal anesthetized rats. In adult rat CMs, Compound A treatment led to a dose-dependent increase in intracellular Ca^2+^ handling efficiency and cell contractility. Intravenous infusion of Compound A in normal rats improved diastolic function, but no improvement was observed in systolic function. This suggests that Compound A may have specific effects on diastolic dysfunction. However, the failure of Compound A to improve systolic function raises questions about its mechanism of action, potentially indicating that the dosing regimen used in the study was not sufficient. Further research is needed to explore Compound A, including animal-based studies in disease models, enhancement of its potency, and optimization of its PK properties.

Istaroxime ([3Z,5α)-3-[(2-Aminoethoxy)imino]androstane-6,17-dione, formerly PST2744) is a steroidal inotrope with a similar mechanism of action as digoxin but less arrhythmic (130). It has a dual activity, inhibiting the membrane Na^+^/K^+^-ATPase pump and stimulating SERCA2a (131). The molecular mechanism of istaroxime involves targeting a pocket formed by the interaction between SERCA2a and PLN or direct binding to specific sites on PLN (132). Clinical studies have proved the positive effects of istaroxime in patients with acute HF (133, 134). A phase 2 trial (NCT04325035) investigated the safety and efficacy of istaroxime and was conducted in patients with acute decompensated HF with persistent hypotension. Although the trial did not reach its primary endpoint, it was found that istaroxime increased systolic blood pressure and improved echocardiogram parameters without increasing the risk of arrhythmias or renal dysfunction (134). As mentioned earlier, the potential efficacy of istaroxime in improving HFpEF has been demonstrated in a preclinical model (38). Recent studies have also suggested that istaroxime may improve overall cardiac function in HFpEF patients by lowing reducing exercise-induced pulmonary capillary wedge pressure (135). Continued studies and clinical trials will provide valuable insight into the therapeutic potential of istaroxime for HFpEF and guide its appropriate use in clinical practice. Efforts to overcome the limitations of istaroxime, such as its very short half-life (<1 h) and lack of SERCA2 selectivity, have led to the development of its metabolite, PST3093 (136). In patients with HF, PST3093 showed a much longer half-life (approximately 9 h) than istaroxime (137). PST3093 does not inhibit the Na^+^/K^+^-ATPase pump, but it was suggested that it enhances SERCA2a activity. PST3093 activity also demonstrated improved efficacy over istaroxime in a rat model of diabetic cardiomyopathy (137). However, the *in vivo* efficacy of PST3093 was assessed only by echocardiography, and optimization of treatment conditions for istaroxime comparison experiments is required. Furthermore an in-depth study is needed on how PST3093 stimulates SERCA2a activity.

Intracellular acting antibodies targeting PLN. Variable domains of the heavy chain antibody (VHH) intrabodies, derived from camelids, have emerged as a novel class of therapeutics due to their small size and ability to target intracellular antigens, offering advantages over traditional antibodies (138). Clinical development of VHH intrabodies, such as caplacizumab for acquired thrombotic thrombocytopenic purpura, is underway (139). Recent research has shown that VHH intrabodies targeting pentameric PLN have potential therapeutic applications for improving cardiac function (140). In this study, the VHH intrabodies targeting pentameric PLN (both non-phosphorylated and phosphorylated forms) were identified, and subsequently, co-immunoprecipitation experiments confirmed the PLN-binding VHH intrabody expression. In a mouse model of dilated cardiomyopathy, the hemodynamic analysis demonstrated improved cardiac contractility after injecting an AAV9 vector carrying a PLN-targeted intrabody (140). The study also identified a specific intrabody that can bind specifically to phosphorylated (Ser16) PLN peptides. However, the phosphorylated PLN specific intrabody showed moderately inhibited Ca^2+^ cycling in adult mouse CM, although its expression level was similar to that of the pentameric PLN targeting intrabody (140). Targeting PLN (particularly distinct from phosphorylated forms) with intrabodies provides an accurate and efficient approach to modifying cellular physiology for the aforementioned PLN mutation-associated cardiomyopathy and other cardiac disease associated with dysregulation of PLN activity.

### 7.5. PDE3A–SERCA2a dissociation

OptF (optimized fragment F) is a small peptide derived from the PDE3A sequences and optimized for binding affinity (141). Early attempts to develop PDE3 inhibitors for the treatment of HF were limited by off-target effects and poor selectivity. However, a new strategy has emerged, targeting PDE3A localized to the SR to develop activators of SERCA2a. In that experimental studies, treating CMs with the specific peptide OptF, which disrupts the binding between PDE3A and SERCA2a, has been shown to enhance the activity of SERCA2a independently of the catalytic activity of PDE3A. Moreover, injection of AAV vectors encoding PDE3A/SERCA2a interfering peptides into mice with pressure-overloaded HF improved animal survival compared to non-treated controls. However, it is noteworthy that this treatment did not have a significant effect on cardiac remodeling. Further studies are required to demonstrate the potential benefit of targeting the PDE3A/SERCA2a interaction using peptides such as OptF for the treatment of HF.

### 7.6. SERCA2a SUMOylation activators

N106 (4-Methoxy-N-[5-(4-methoxyphenyl)-1,3,4-oxadiazol-2-yl-2-benzothiazolamine]) is a first-in-class SERCA2a SUMOylation activator. N106 stimulates SUMO-activating enzyme E1 to activate intrinsic SERCA2a SUMOylation, enhancing SERCA2a function and improving cardiac contractility in a HF mouse model (120). Studies on ADME, mouse PK, and NCI-60 tests have supported the pharmacological properties and off-target effects of N106 (120). The therapeutic efficacy of N106 is currently being evaluated in animals with DMD.

Ginsenosides Rg3 (Rg3) are a group of steroidal saponins found in ginseng with pharmacologically active abilities. In a recent study, the potential therapeutic effects and mechanisms of Rg3 were evaluated in a pressure-overload-induced HF mouse model (142). Treatment with Rg3 significantly improved heart function in HF mice with an increase in SUMOylation levels and SERCA2a activity (142). Meanwhile, the positive effects of Rg3 in HF were limited in SUMO1 knockout mice, supporting the mechanism of Rg3 action (142). Also, Rg3 treatment induced Ubc9 expression, a SUMO conjugating enzyme E2 in HF mice, suggesting the presence of additional molecular mechanisms involved in cardiac SUMOylation.

Luteolin (3′,4′,5,7-tetrahydroxy flavone) is the most common flavonoid in plants and has biological activities and PK properties (143). Numerous animal studies have demonstrated the cardioprotective properties of luteolin, which involve the partial modulation of SERCA2a through different mechanisms. One mechanism involves the upregulation of the Sp1 transcription factor. Luteolin has been shown to enhance the expression of Sp1, which in turn leads to an increased level of SERCA2a (144). Another mechanism involves the upregulation of SUMO1. Luteolin has been reported to increase the levels of SUMO, which promotes the SUMOylation of SERCA2a (145). This modification enhances the activity and stability of SERCA2a. Furthermore, luteolin has been found to increase the phosphorylation of PLN through the inhibition of the p38 MAPK signaling pathway (145). However, clinical studies focused on luteolin were limited.

### 7.7. SERCA2a deacetylation enhancers

β-lapachone (3,4-dihydro-2,2-dimethyl-2H-naphthol[1,2-b]pyran-5,6-dione, ARQ761 in clinical form) is an antitumor drug currently undergoing clinical development. It has been shown to directly kill cancer cells that overexpress NADH-quinone oxidoreductase 1 (146). In addition to its antitumor effects, β-lapachone has been found to stimulate SIRT1 deacetylase activity by modulating cellular NAD^+^/NADH ratios under pathogenic cardiac conditions, thereby regulating SERCA2a acetylation and deacetylation (104). Pharmacological activation of SIRT1 through β-lapachone treatment can facilitate the restoration of SERCA2a activity by promoting deacetylation *in vivo* (104). Further studies strongly suggest that SIRT1 activators, such as resveratrol, can potentially treat heart disease, including diabetic cardiomyopathy and HF, by enhancing SERCA2a (147–149).

Resveratrol (3,4′,5,-trihydroxystilbene), a natural polyphenolic compound, is a potent activator of SIRT1 and cardioprotective properties under conditions where SIRT1 is depleted. In a mouse model of chronic type 1 diabetes, a study showed that streptozotocin (STZ) administration led to a progressive decline in cardiac function accompanied by decreased levels of SERCA2a and SIRT1 proteins (147). However, the treatment of resveratrol had a significant positive impact on both SERCA2a expression and cardiac function (147). In high glucose conditions, resveratrol counteracted the suppression of the SERCA2a promoter activity in cultured CMs, and this protective effect relied on the activation of SIRT1. Additionally, mice lacking one copy of the SIRT1 gene showed increased sensitivity to STZ-induced decline in SERCA2a mRNA (147). Resveratrol was able to prevent pressure-overload-induced alternation of multiple key Ca^2+^ handling proteins, including the SERCA2a pump, PLN, NCX, and RyR2 in rats (148). In that study, resveratrol effectively prevented the development of cardiac dysfunction, chamber dilation, and fibrosis in aortic-banded rats, with the timing of treatment being a critical factor for achieving regression of HT (148). Clinical studies estimating the safety and efficacy of resveratrol for the treatment of HF are limited. However, a small cohort study conducted in a single center reported positive outcomes in NYHA class II-III HF patients with reduced ejection fraction demonstrating improvements in cardiac function, exercise capacity, and quality of life, along with reductions in cardiac biomarkers and inflammatory cytokines (149).The treatment duration was relatively short (12 weeks), and the lack of placebo groups in the study requires further research in this area.

## 8. Discussion

Based on current evidence, therapies targeting the restoration or upregulation of SERCA2a have the potential to effectively manage HF across diverse underlying causes, particularly in the chronic phase of diseases, by improving both systolic and diastolic function, offering significant advantages for patients. However, it should be is considered that excessive upregulation of SERCA2a, as observed in animal studies, may have negative effects. For instance, overexpression of the skeletal muscle isoform of SERCA (SERCA1a) in hypertrophic rat hearts resulted in weakened cardiac function (150). This detrimental effect may be due to a potential limitation of Ca^2+^ transient amplitude in the CMs caused by high expression of SERCA1 protein (151), which exhibits faster Ca^2+^ transport kinetics properties compared to SERCA2a. Furthermore, compensatory enhanced SR-cytosol Ca^2+^ cycling in the early stage of HT suggests that the efficacy of SERCA2a gene therapy may be maximized during late-stage remodeling (152). However, these findings are derived from small animal studies and should be interpreted with caution, as they may not directly apply to humans.

In addition to targeting SERCA2a directly, novel approach are being explored to enhance the clinical efficiency of SERCA2a therapy. One such approach involves combining SERCA2a gene therapy with drugs targeting PTM as a fine-tuning mechanism in the treatment of various patients. The potential synergetic effects of SERCA2a and SUMO1 have been identified in a pig model of HF (115). Further studies on combination treatments with SERCA2a gene therapy and PTM modulators, such as SUMO1 molecules or N106, are needed to advance this perspective.

MiRNAs targeting SERCA2a have shown promise as potential therapeutic agents. However, there are no miR-based drugs have reached phase 3 clinical trials yet, and careful consideration is needed for problems related to on/off-target side effects and safety issues arising from targeting multiple genes. However, with the rapid advancement of RNA therapeutics, there are opportunities for the future development of miRNA-based drugs.

Antibody and peptide inhibitors represent promising approaches for targeted and specific modulation of SERCA2a activity in the preclinical setting. These inhibitors, although still in the early stage of development, can be designed to address specific dysfunctions or regulatory mechanisms associated with SERCA2a in the context of HF.

## 9. Future directions

Understanding the complex mechanisms underlying SERCA2a expression and activity regulation in pathological settings is a focal point of numerous studies in the field. Developing isoform- and tissue-specific modulators with minimizing off-target effects represents a challenging yet pivotal goal, as it will significantly advance SERCA2a-targeted therapy. Therefore, it is vital to comprehend the long-term effects of SERCA2a modulators, including their impact on cardiac remodeling, arrhythmia, and overall outcomes. Moreover, investigating potential synergies and safety profiles when combining SERCA2a modulators with other therapies, such as beta-blockers or angiotensin-converting enzyme inhibitors, remains a complex challenge. By employing diverse approaches like gene therapy, pharmacological agents, miRNAs, antibodies, or peptide inhibitors, to target SERCA2a modulators, we can potentially enhance SERCA2a levels and activity in a more precise and controlled manner. This personalized strategy holds promise for optimizing the SERCA2a function. Rigorous research and well-designed clinical trials are imperative to establish these therapeutic approaches’ efficacy, safety, and long-term benefits in treating HF.

## References

[1] Disease GBD Injury I, Prevalence C. Global, regional, and national incidence, prevalence, and years lived with disability for 354 diseases and injuries for 195 countries and territories, 1990–2017: a systematic analysis for the global burden of disease study 2017. Lancet. (2018) 392(10159):1789–858. 10.1016/S0140-6736(18)32279-7 30496104 PMC6227754

[2] Groenewegen A Rutten FH Mosterd A Hoes AW. Epidemiology of heart failure. Eur J Heart Fail. (2020) 22(8):1342–56. 10.1002/ejhf.1858 32483830 PMC7540043

[3] Hasenfuss G Mulieri LA Leavitt BJ Allen PD Haeberle JR Alpert NR. Alteration of contractile function and excitation-contraction coupling in dilated cardiomyopathy. Circ Res. (1992) 70(6):1225–32. 10.1161/01.res.70.6.1225 1576741

[4] Beuckelmann DJ Näbauer M Erdmann E. Intracellular calcium handling in isolated ventricular myocytes from patients with terminal heart failure. Circulation. (1992) 85(3):1046–55. 10.1161/01.cir.85.3.1046 1311223

[5] Pieske B Sütterlin M Schmidt-Schweda S Minami K Meyer M Olschewski M Diminished post-rest potentiation of contractile force in human dilated cardiomyopathy. Functional evidence for alterations in intracellular Ca^2+^ handling. J Clin Invest. (1996) 98(3):764–76. 10.1172/JCI118849 8698869 PMC507487

[6] Gwathmey JK Copelas L MacKinnon R Schoen FJ Feldman MD Grossman W Abnormal intracellular calcium handling in myocardium from patients with end-stage heart failure. Circ Res. (1987) 61(1):70–6. 10.1161/01.res.61.1.70 3608112

[7] Mercadier JJ Lompré AM Duc P Boheler KR Fraysse JB Wisnewsky C Altered sarcoplasmic reticulum Ca2(+)-ATPase gene expression in the human ventricle during end-stage heart failure. J Clin Invest. (1990) 85(1):305–9. 10.1172/JCI114429 2136864 PMC296420

[8] Arai M Alpert NR MacLennan DH Barton P Periasamy M. Alterations in sarcoplasmic reticulum gene expression in human heart failure. A possible mechanism for alterations in systolic and diastolic properties of the failing myocardium. Circ Res. (1993) 72(2):463–9. 10.1161/01.res.72.2.463 8418995

[9] Takahashi T Allen PD Izumo S. Expression of A-, B-, and C-type natriuretic peptide genes in failing and developing human ventricles. Correlation with expression of the Ca(2+)-ATPase gene. Circ Res. (1992) 71(1):9–17. 10.1161/01.res.71.1.9 1535030

[10] Bers DM. Cardiac excitation-contraction coupling. Nature. (2002) 415(6868):198–205. 10.1038/415198a 11805843

[11] Bassani JW Bassani RA Bers DM. Relaxation in rabbit and rat cardiac cells: species-dependent differences in cellular mechanisms. J Physiol. (1994) 476(2):279–93. 10.1113/jphysiol.1994.sp020130 8046643 PMC1160440

[12] Hove-Madsen L Bers DM. Sarcoplasmic reticulum Ca2+ uptake and thapsigargin sensitivity in permeabilized rabbit and rat ventricular myocytes. Circ Res. (1993) 73(5):820–8. 10.1161/01.res.73.5.820 8403253

[13] Periasamy M Reed TD Liu LH Ji Y Loukianov E Paul RJ Impaired cardiac performance in heterozygous mice with a null mutation in the sarco(endo)plasmic reticulum Ca^2+^-ATPase isoform 2 (SERCA2) gene. J Biol Chem. (1999) 274(4):2556–62. 10.1074/jbc.274.4.2556 9891028

[14] Huke S Liu LH Biniakiewicz D Abraham WT Periasamy M. Altered force-frequency response in non-failing hearts with decreased SERCA pump-level. Cardiovasc Res. (2003) 59(3):668–77. 10.1016/s0008-6363(03)00436-x 14499868

[15] Andersson KB Birkeland JA Finsen AV Louch WE Sjaastad I Wang Y Moderate heart dysfunction in mice with inducible cardiomyocyte-specific excision of the Serca2 gene. J Mol Cell Cardiol. (2009) 47(2):180–7. 10.1016/j.yjmcc.2009.03.013 19328205

[16] Louch WE Hougen K Mørk HK Swift F Aronsen JM Sjaastad I Sodium accumulation promotes diastolic dysfunction in end-stage heart failure following Serca2 knockout. J Physiol. (2010) 588(Pt 3):465–78. 10.1113/jphysiol.2009.183517 20008467 PMC2825611

[17] Studer R Reinecke H Bilger J Eschenhagen T Böhm M Hasenfuss G Gene expression of the cardiac Na(+)-Ca2+ exchanger in end-stage human heart failure. Circ Res. (1994) 75(3):443–53. 10.1161/01.res.75.3.443 8062418

[18] Hasenfuss G Reinecke H Studer R Meyer M Pieske B Holtz J Relation between myocardial function and expression of sarcoplasmic reticulum Ca(2+)-ATPase in failing and nonfailing human myocardium. Circ Res. (1994) 75(3):434–42. 10.1161/01.res.75.3.434 8062417

[19] Venetucci LA Trafford AW O'Neill SC Eisner DA. The sarcoplasmic reticulum and arrhythmogenic calcium release. Cardiovasc Res. (2008) 77(2):285–92. 10.1093/cvr/cvm009 18006483

[20] Lyon AR MacLeod KT Zhang Y Garcia E Kanda GK Lab MJ Loss of T-tubules and other changes to surface topography in ventricular myocytes from failing human and rat heart. Proc Natl Acad Sci U S A. (2009) 106(16):6854–9. 10.1073/pnas.0809777106 19342485 PMC2672472

[21] O'Neill SC Miller L Hinch R Eisner DA. Interplay between SERCA and sarcolemmal Ca2+ efflux pathways controls spontaneous release of Ca2+ from the sarcoplasmic reticulum in rat ventricular myocytes. J Physiol. (2004) 559(Pt 1):121–8. 10.1113/jphysiol.2003.058917 15194743 PMC1665077

[22] Fernandez-Tenorio M Niggli E. Stabilization of Ca^2+^ signaling in cardiac muscle by stimulation of SERCA. J Mol Cell Cardiol. (2018) 119:87–95. 10.1016/j.yjmcc.2018.04.015 29715473

[23] van der Zwaag PA van Rijsingen IA Asimaki A Jongbloed JD van Veldhuisen DJ Wiesfeld AC Phospholamban R14del mutation in patients diagnosed with dilated cardiomyopathy or arrhythmogenic right ventricular cardiomyopathy: evidence supporting the concept of arrhythmogenic cardiomyopathy. Eur J Heart Fail. (2012) 14(11):1199–207. 10.1093/eurjhf/hfs119 22820313 PMC3475434

[24] Haghighi K Kolokathis F Gramolini AO Waggoner JR Pater L Lynch RA A mutation in the human phospholamban gene, deleting arginine 14, results in lethal, hereditary cardiomyopathy. Proc Natl Acad Sci U S A. (2006) 103(5):1388–93. 10.1073/pnas.0510519103 16432188 PMC1360586

[25] del Monte F Williams E Lebeche D Schmidt U Rosenzweig A Gwathmey JK Improvement in survival and cardiac metabolism after gene transfer of sarcoplasmic reticulum Ca(2+)-ATPase in a rat model of heart failure. Circulation. (2001) 104(12):1424–9. 10.1161/hc3601.095574 11560860 PMC1249503

[26] Kawase Y Ly HQ Prunier F Lebeche D Shi Y Jin H Reversal of cardiac dysfunction after long-term expression of SERCA2a by gene transfer in a pre-clinical model of heart failure. J Am Coll Cardiol. (2008) 51(11):1112–9. 10.1016/j.jacc.2007.12.014 18342232

[27] Byrne MJ Power JM Preovolos A Mariani JA Hajjar RJ Kaye DM. Recirculating cardiac delivery of AAV2/1SERCA2a improves myocardial function in an experimental model of heart failure in large animals. Gene Ther. (2008) 15(23):1550–7. 10.1038/gt.2008.120 18650850

[28] del Monte F Harding SE Schmidt U Matsui T Kang ZB Dec GW Restoration of contractile function in isolated cardiomyocytes from failing human hearts by gene transfer of SERCA2a. Circulation. (1999) 100(23):2308–11. 10.1161/01.cir.100.23.2308 10587333 PMC1249502

[29] Packer M Carver JR Rodeheffer RJ Ivanhoe RJ DiBianco R Zeldis SM Effect of oral milrinone on mortality in severe chronic heart failure. The PROMISE study research group. N Engl J Med. (1991) 325(21):1468–75. 10.1056/NEJM199111213252103 1944425

[30] Motloch LJ Cacheux M Ishikawa K Xie C Hu J Aguero J Primary effect of SERCA 2a gene transfer on conduction reserve in chronic myocardial infarction. J Am Heart Assoc. (2018) 7(18):e009598. 10.1161/JAHA.118.009598 30371209 PMC6222964

[31] Prunier F Kawase Y Gianni D Scapin C Danik SB Ellinor PT Prevention of ventricular arrhythmias with sarcoplasmic reticulum Ca^2+^ ATPase pump overexpression in a porcine model of ischemia reperfusion. Circulation. (2008) 118(6):614–24. 10.1161/CIRCULATIONAHA.108.770883 18645052

[32] del Monte F Hajjar RJ Harding SE. Overwhelming evidence of the beneficial effects of SERCA gene transfer in heart failure. Circ Res. (2001) 88(11):E66–7. 10.1161/hh1101.092004 11397790

[33] Studeli R Jung S Mohacsi P Perruchoud S Castiglioni P Wenaweser P Diastolic dysfunction in human cardiac allografts is related with reduced SERCA2a gene expression. Am J Transplant. (2006) 6(4):775–82. 10.1111/j.1600-6143.2006.01241.x 16539635

[34] Lim CC Liao R Varma N Apstein CS. Impaired lusitropy-frequency in the aging mouse: role of Ca(2+)-handling proteins and effects of isoproterenol. Am J Physiol. (1999) 277(5):H2083–90. 10.1152/ajpheart.1999.277.5.H2083 10564164

[35] Schmidt U del Monte F Miyamoto MI Matsui T Gwathmey JK Rosenzweig A Restoration of diastolic function in senescent rat hearts through adenoviral gene transfer of sarcoplasmic reticulum Ca(2+)-ATPase. Circulation. (2000) 101(7):790–6. 10.1161/01.cir.101.7.790 10683354

[36] Cain BS Meldrum DR Joo KS Wang JF Meng X Cleveland JC Jr Human SERCA2a levels correlate inversely with age in senescent human myocardium. J Am Coll Cardiol. (1998) 32(2):458–67. 10.1016/s0735-1097(98)00233-2 9708476

[37] Belke DD Swanson EA Dillmann WH. Decreased sarcoplasmic reticulum activity and contractility in diabetic db/db mouse heart. Diabetes. (2004) 53(12):3201–8. 10.2337/diabetes.53.12.3201 15561951

[38] Torre E Arici M Lodrini AM Ferrandi M Barassi P Hsu SC SERCA2a Stimulation by istaroxime improves intracellular Ca2+ handling and diastolic dysfunction in a model of diabetic cardiomyopathy. Cardiovasc Res. (2022) 118(4):1020–32. 10.1093/cvr/cvab123 33792692 PMC8930067

[39] Wankerl M Boheler KR Fiszman MY Schwartz K. Molecular cloning and analysis of the human cardiac sarco(endo)plasmic reticulum Ca(2+)-ATPase (SERCA2) gene promoter. J Mol Cell Cardiol. (1996) 28(10):2139–50. 10.1006/jmcc.1996.0206 8930809

[40] Zarain-Herzberg A Alvarez-Fernandez G. Sarco(endo)plasmic reticulum Ca^2+^-ATPase-2 gene: structure and transcriptional regulation of the human gene. ScientificWorldJournal. (2002) 2:1469–83. 10.1100/tsw.2002.228 12805933 PMC6009281

[41] Sack MN Disch DL Rockman HA Kelly DP. A role for Sp and nuclear receptor transcription factors in a cardiac hypertrophic growth program. Proc Natl Acad Sci U S A. (1997) 94(12):6438–43. 10.1073/pnas.94.12.6438 9177236 PMC21068

[42] Ronkainen VP Skoumal R Tavi P. Hypoxia and HIF-1 suppress SERCA2a expression in embryonic cardiac myocytes through two interdependent hypoxia response elements. J Mol Cell Cardiol. (2011) 50(6):1008–16. 10.1016/j.yjmcc.2011.02.017 21382378

[43] Williams AL Walton CB MacCannell KA Avelar A Shohet RV. HIF-1 regulation of miR-29c impairs SERCA2 expression and cardiac contractility. Am J Physiol Heart Circ Physiol. (2019) 316(3):H554–H65. 10.1152/ajpheart.00617.2018 30575439 PMC6459310

[44] Tsai CT Wu CK Lee JK Chang SN Kuo YM Wang YC TNF-alpha down-regulates sarcoplasmic reticulum Ca(2)(+) ATPase expression and leads to left ventricular diastolic dysfunction through binding of NF-kappaB to promoter response element. Cardiovasc Res. (2015) 105(3):318–29. 10.1093/cvr/cvv00 25712896

[45] Angrisano T Schiattarella GG Keller S Pironti G Florio E Magliulo F Epigenetic switch at atp2a2 and myh7 gene promoters in pressure overload-induced heart failure. PLoS One. (2014) 9(9):e106024. 10.1371/journal.pone.0106024 25181347 PMC4152141

[46] Liu L Zhao W Liu J Gan Y Liu L Tian J. Epigallocatechin-3 gallate prevents pressure overload-induced heart failure by up-regulating SERCA2a via histone acetylation modification in mice. PLoS One. (2018) 13(10):e0205123. 10.1371/journal.pone.0205123 30286210 PMC6171916

[47] Stenzig J Hirt MN Loser A Bartholdt LM Hensel JT Werner TR DNA methylation in an engineered heart tissue model of cardiac hypertrophy: common signatures and effects of DNA methylation inhibitors. Basic Res Cardiol. (2016) 111(1):9. 10.1007/s00395-015-0528-z 26680771

[48] Oh JG Jang SP Yoo J Lee MA Lee SH Lim T Role of the PRC2-Six1-miR-25 signaling axis in heart failure. J Mol Cell Cardiol. (2019) 129:58–68. 10.1016/j.yjmcc.2019.01.017 30771307

[49] Zang B Lin H Xiao J Lu Y Luo X Li B The muscle-specific microRNA miR-1 regulates cardiac arrhythmogenic potential by targeting GJA1 and KCNJ2. Nat Med. (2007) 13(4):486–91. 10.1038/nm1569 17401374

[50] Carè A Catalucci D Felicetti F Bonci D Addario A Gallo P MicroRNA-133 controls cardiac hypertrophy. Nat Med. (2007) 13(5):613–8. 10.1038/nm1582 17468766

[51] van Rooij E Sutherland LB Liu N Williams AH McAnally J Gerard RD A signature pattern of stress-responsive microRNAs that can evoke cardiac hypertrophy and heart failure. Proc Natl Acad Sci U S A. (2006) 103(48):18255–60. 10.1073/pnas.0608791103 17108080 PMC1838739

[52] Mendell JT. Miriad roles for the miR-17-92 cluster in development and disease. Cell. (2008) 133(2):217–22. 10.1016/j.cell.2008.04.001 18423194 PMC2732113

[53] Wahlquist C Jeong D Rojas-Munoz A Kho C Lee A Mitsuyama S Inhibition of miR-25 improves cardiac contractility in the failing heart. Nature. (2014) 508(7497):531–5. 10.1038/nature13073 24670661 PMC4131725

[54] Jeong D Yoo J Lee P Kepreotis SV Lee A Wahlquist C miR-25 tough decoy enhances cardiac function in heart failure. Mol Ther. (2018) 26(3):718–29. 10.1016/j.ymthe.2017.11.014 29273502 PMC5910658

[55] Dirkx E Gladka MM Philippen LE Armand AS Kinet V Leptidis S Nfat and miR-25 cooperate to reactivate the transcription factor Hand2 in heart failure. Nat Cell Biol. (2013) 15(11):1282–93. 10.1038/ncb2866 24161931

[56] Bush EW van Rooij E. miR-25 in heart failure. Circ Res. (2014) 115(7):610–2. 10.1161/CIRCRESAHA.114.304909 25214573

[57] Li C Li X Gao X Zhang R Zhang Y Liang H MicroRNA-328 as a regulator of cardiac hypertrophy. Int J Cardiol. (2014) 173(2):268–76. 10.1016/j.ijcard.2014.02.035 24631114

[58] Lei Z Wahlquist C El Azzouzi H Deddens JC Kuster D van Mil A miR-132/212 impairs cardiomyocytes contractility in the failing heart by suppressing SERCA2a. Front Cardiovasc Med. (2021) 8:592362. 10.3389/fcvm.2021.592362 33816571 PMC8017124

[59] Taubel J Hauke W Rump S Viereck J Batkai S Poetzsch J Novel antisense therapy targeting microRNA-132 in patients with heart failure: results of a first-in-human phase 1b randomized, double-blind, placebo-controlled study. Eur Heart J. (2021) 42(2):178–88. 10.1093/eurheartj/ehaa898 33245749 PMC7954267

[60] Lu D Thum T. RNA-based diagnostic and therapeutic strategies for cardiovascular disease. Nat Rev Cardiol. (2019) 16(11):661–74. 10.1038/s41569-019-0218-x 31186539

[61] Askarian-Amiri ME Crawford J French JD Smart CE Smith MA Clark MB SNORD-host RNA Zfas1 is a regulator of mammary development and a potential marker for breast cancer. RNA. (2011) 17(5):878–91. 10.1261/rna.2528811 21460236 PMC3078737

[62] Zhang Y Jiao L Sun L Li Y Gao Y Xu C LncRNA ZFAS1 as a SERCA2a inhibitor to cause intracellular Ca(2+) overload and Contractile dysfunction in a mouse model of myocardial infarction. Circ Res. (2018) 122(10):1354–68. 10.1161/CIRCRESAHA.117.312117 29475982 PMC5959220

[63] Li J Sun D Pu W Wang J Peng Y. Circular RNAs cancer treatment. Trends Cancer. (2020) 6(4):319–36. 10.1016/j.trecan.2020.01.012 32209446

[64] Han D Wang Y Wang Y Dai X Zhou T Chen J The tumor-suppressive human circular RNA CircITCH sponges miR-330-5p to ameliorate doxorubicin-induced cardiotoxicity through upregulating SIRT6, survivin, and SERCA2a. Circ Res. (2020) 127(4):e108–e25. 10.1161/CIRCRESAHA.119.316061 32392088

[65] Wegener AD Simmenrman HK Lindemann JP Jones LR. Phospholamban phosphorylation in intact ventricles. Phosphorylation of serine 16 and threonine 17 in response to beta-adrenergic stimulation. J Biol Chem. (1989) 264(19):11468–74. 10.1016/S0021-9258(18)60487-9 2544595

[66] Karim CB Zhang Z Howard EC Torgersen KD Thomas DD. Phosphorylation-dependent conformational switch in spin-labeled phospholamban bound to SERCA. J Mol Biol. (2006) 358(4):1032–40. 10.1016/j.jmb.2006.02.051 16574147

[67] MacDougall LK Jones LR Cohen P. Identification of the major protein phosphatases in mammalian cardiac muscle which dephosphorylate phospholamban. Eur J Biochem. (1991) 196(3):725–34. 10.1111/j.1432-1033.1991.tb15871.x 1849481

[68] Lygren B Carlson CR Santamaria K Lissandron V McSorley T Litzenberg J AKAP complex regulates Ca2+ re-uptake into heart sarcoplasmic reticulum. EMBO Rep. (2007) 8(11):1061–7. 10.1038/sj.embor.7401081 17901878 PMC2247390

[69] Carlson CR Aronsen JM Bergan-Dahl A Moutty MC Lunde M Lunde PK AKAP18δ anchors and regulates CaMKII activity at phospholamban-SERCA2 and RYR. Circ Res. (2022) 130(1):27–44. 10.1161/CIRCRESAHA.120.317976 34814703 PMC9500498

[70] Singh A Redden JM Kapiloff MS Dodge-Kafka KL. The large isoforms of A-kinase anchoring protein 18 mediate the phosphorylation of inhibitor-1 by protein kinase A and the inhibition of protein phosphatase 1 activity. Mol Pharmacol. (2011) 79(3):533–40. 10.1124/mol.110.065425 21149637 PMC3061358

[71] del Monte F Harding SE Dec GW Gwathmey JK Hajjar RJ. Targeting phospholamban by gene transfer in human heart failure. Circulation. (2002) 105(8):904–7. 10.1161/hc0802.105564 11864915 PMC1249505

[72] Minamisawa S Hoshijima M Chu G Ward CA Frank K Gu Y Chronic phospholamban-sarcoplasmic reticulum calcium ATPase interaction is the critical calcium cycling defect in dilated cardiomyopathy. Cell. (1999) 99(3):313–22. 10.1016/s0092-8674(00)81662-1 10555147

[73] Schwinger RH Munch G Bolck B Karczewski P Krause EG Erdmann E. Reduced Ca(2+)-sensitivity of SERCA 2a in failing human myocardium due to reduced serin-16 phospholamban phosphorylation. J Mol Cell Cardiol. (1999) 31(3):479–91. 10.1006/jmcc.1998.0897 10198180

[74] El-Armouche A Pamminger T Ditz D Zolk O Eschenhagen T. Decreased protein and phosphorylation level of the protein phosphatase inhibitor-1 in failing human hearts. Cardiovasc Res. (2004) 61(1):87–93. 10.1016/j.cardiores.2003 14732205

[75] Carr AN Schmidt AG Suzuki Y del Monte F Sato Y Lanner C Type 1 phosphatase, a negative regulator of cardiac function. Mol Cell Biol. (2002) 22(12):4124–35. 10.1128/MCB.22.12.4124-4135.2002 12024026 PMC133876

[76] Oh JG Kim J Jang SP Nguen M Yang DK Jeong D Decoy peptides targeted to protein phosphatase 1 inhibit dephosphorylation of phospholamban in cardiomyocytes. J Mol Cell Cardiol. (2013) 56:63–71. 10.1016/j.yjmcc.2012.12.005 23262438

[77] Pathak A del Monte F Zhao W Schultz JE Lorenz JN Bodi I Enhancement of cardiac function and suppression of heart failure progression by inhibition of protein phosphatase 1. Circ Res. (2005) 96(7):756–66. 10.1161/01.RES.0000161256.85833.fa 15746443

[78] Watanabe S Ishikawa K Fish K Oh JG Motloch LJ Kohlbrenner E Protein phosphatase inhibitor-1 gene therapy in a swine model of nonischemic heart failure. J Am Coll Cardiol. (2017) 70(14):1744–56. 10.1016/j.jacc.2017.08.013 28958332 PMC5807083

[79] Schmitt JP Kamisago M Asahi M Li GH Ahmad F Mende U Dilated cardiomyopathy and heart failure caused by a mutation in phospholamban. Science. (2003) 299(5611):1410–3. 10.1126/science.1081578 12610310

[80] Haghighi K Kolokathis F Pater L Lynch RA Asahi M Gramolini AO Human phospholamban null results in lethal dilated cardiomyopathy revealing a critical difference between mouse and human. J Clin Invest. (2003) 111(6):869–76. 10.1172/JCI17892 12639993 PMC153772

[81] Nelson BR Makarewich CA Anderson DM Winders BR Troupes CD Wu F A peptide encoded by a transcript annotated as long noncoding RNA enhances SERCA activity in muscle. Science. (2016) 351(6270):271–5. 10.1126/science.aad4076 26816378 PMC4892890

[82] Singh DR Dalton MP Cho EE Pribadi MP Zak TJ Seflova J Newly discovered micropeptide regulators of SERCA form oligomers but bind to the pump as monomers. J Mol Biol. (2019) 431(22):4429–43. 10.1016/j.jmb.2019.07.037 31449798 PMC6885550

[83] Fisher ME Bovo E Aguayo-Ortiz R Cho EE Pribadi MP Dalton MP Dwarf open Reading frame (DWORF) is a direct activator of the sarcoplasmic reticulum calcium pump SERCA. eLife. (2021) 10:e65545. 10.7554/eLife.65545 34075877 PMC8203291

[84] Cleary SR Fang X Cho EE Pribadi MP Seflova J Beach JR Inhibitory and stimulatory micropeptides preferentially bind to different conformations of the cardiac calcium pump. J Biol Chem. (2022) 298(7):102060. 10.1016/j.jbc.2022.102060 35605666 PMC9218510

[85] Makarewich CA Munir AZ Schiattarella GG Bezprozvannaya S Raguimova ON Cho EE The DWORF micropeptide enhances contractility and prevents heart failure in a mouse model of dilated cardiomyopathy. eLife. (2018) 7:e38319. 10.7554/eLife.38319 30299255 PMC6202051

[86] Makarewich CA Bezprozvannaya S Gibson AM Bassel-Duby R Olson EN. Gene therapy with the DWORF micropeptide attenuates cardiomyopathy in mice. Circ Res. (2020) 127(10):1340–2. 10.1161/CIRCRESAHA.120.317156 32878549 PMC7581560

[87] Morales ED Yue Y Watkins TB Han J Pan X Gibson AM Dwarf open reading frame (DWORF) gene therapy ameliorated duchenne muscular dystrophy cardiomyopathy in aged mdx mice. J Am Heart Assoc. (2023) 12(3):e027480. 10.1161/JAHA.122.027480 36695318 PMC9973626

[88] Weiss JL Frederiksen JW Weisfeldt ML. Hemodynamic determinants of the time-course of fall in canine left ventricular pressure. J Clin Invest. (1976) 58(3):751–60. 10.1172/JCI108522 956400 PMC333234

[89] Arvanitis DA Vafiadaki E Fan GC Mitton BA Gregory KN Del Monte F Histidine-rich ca-binding protein interacts with sarcoplasmic reticulum ca-ATPase. Am J Physiol Heart Circ Physiol. (2007) 293(3):H1581–9. 10.1152/ajpheart.00278.2007 17526652

[90] Kiewitz R Acklin C Schafer BW Maco B Uhrik B Wuytack F Ca^2+^-dependent interaction of S100A1 with the sarcoplasmic reticulum Ca^2+^-ATPase2a and phospholamban in the human heart. Biochem Biophys Res Commun. (2003) 306(2):550–7. 10.1016/s0006-291x(03)00987-2 12804600

[91] Arvanitis DA Sanoudou D Kolokathis F Vafiadaki E Papalouka V Kontrogianni-Konstantopoulos A The Ser96Ala variant in histidine-rich calcium-binding protein is associated with life-threatening ventricular arrhythmias in idiopathic dilated cardiomyopathy. Eur Heart J. (2008) 29(20):2514–25. 10.1093/eurheartj/ehn328 18617481 PMC2567024

[92] Pleger ST Shan C Ksienzyk J Bekeredjian R Boekstegers P Hinkel R Cardiac AAV9-S100A1 gene therapy rescues post-ischemic heart failure in a preclinical large animal model. Sci Transl Med. (2011) 3(92):92ra64. 10.1126/scitranslmed.3002097 21775667 PMC4095769

[93] Brinks H Rohde D Voelkers M Qiu G Pleger ST Herzog N S100a1 genetically targeted therapy reverses dysfunction of human failing cardiomyocytes. J Am Coll Cardiol. (2011) 58(9):966–73. 10.1016/j.jacc.2011.03.054 21851887 PMC3919460

[94] Soltani L Kheirouri S Enamzadeh E. Elevated serum levels of S100A1 and zinc alpha2-glycoprotein in patients with heart failure. Nutr Metab Cardiovasc Dis. (2021) 31(1):162–8. 10.1016/j.numecd.2020.07.029 33257194

[95] Cuffe MS Califf RM Jr AK Benza R Bourge R Colucci WS Short-term intravenous milrinone for acute exacerbation of chronic heart failure: a randomized controlled trial. JAMA. (2002) 287(12):1541–7. 10.1001/jama.287.12.1541 11911756

[96] Sun B Li H Shakur Y Hensley J Hockman S Kambayashi J Role of phosphodiesterase type 3A and 3B in regulating platelet and cardiac function using subtype-selective knockout mice. Cell Signal. (2007) 19(8):1765–71. 10.1016/j.cellsig.2007.03.012 17482796

[97] Beca S Ahmad F Shen W Liu J Makary S Polidovitch N Phosphodiesterase type 3A regulates basal myocardial contractility through interacting with sarcoplasmic reticulum calcium ATPase type 2a signaling complexes in mouse heart. Circ Res. (2013) 112(2):289–97. 10.1161/CIRCRESAHA.111.300003 23168336 PMC3579621

[98] Ahmad F Shen W Vandeput F Szabo-Fresnais N Krall J Degerman E Regulation of sarcoplasmic reticulum Ca^2+^ ATPase 2 (SERCA2) activity by phosphodiesterase 3A (PDE3A) in human myocardium: phosphorylation-dependent interaction of PDE3A1 with SERCA2. J Biol Chem. (2015) 290(11):6763–76. 10.1074/jbc.M115.638585 25593322 PMC4358103

[99] Dhalla AK Hill MF Singal PK. Role of oxidative stress in transition of hypertrophy to heart failure. J Am Coll Cardiol. (1996) 28(2):506–14. 10.1016/0735-1097(96)00140-4 8800132

[100] Lancel S Qin F Lennon SL Zhang J Tong X Mazzini MJ Oxidative posttranslational modifications mediate decreased SERCA activity and myocyte dysfunction in galphaq-overexpressing mice. Circ Res. (2010) 107(2):228–32. 10.1161/CIRCRESAHA.110.217570 20508180 PMC2909347

[101] Qin F Siwik DA Lancel S Zhang J Kuster GM Luptak I Hydrogen peroxide-mediated SERCA cysteine 674 oxidation contributes to impaired cardiac myocyte relaxation in senescent mouse heart. J Am Heart Assoc. (2013) 2(4):e000184. 10.1161/JAHA.113.000184 23963753 PMC3828801

[102] Radi R. Protein tyrosine nitration: biochemical mechanisms and structural basis of functional effects. Acc Chem Res. (2013) 46(2):550–9. 10.1021/ar300234c 23157446 PMC3577981

[103] Knyushko TV Sharov VS Williams TD Schoneich C Bigelow DJ. 3-Nitrotyrosine Modification of SERCA2a in the aging heart: a distinct signature of the cellular redox environment. Biochemistry. (2005) 44(39):13071–81. 10.1021/bi051226n 16185075

[104] Gorski PA Jang SP Jeong D Lee A Lee P Oh JG Role of SIRT1 in modulating acetylation of the sarco-endoplasmic Reticulum Ca(2+)-ATPase in heart failure. Circ Res. (2019) 124(9):e63–80. 10.1161/CIRCRESAHA.118.313865 30786847 PMC6483854

[105] Gorski PA Lee A Lee P Oh JG Vangheluwe P Ishikawa K Identification and characterization of p300-mediated lysine residues in cardiac SERCA2a. Int J Mol Sci. (2023) 24(4):3502. 10.3390/ijms24043502 36834924 PMC9959367

[106] Zhang Y Inaba K. Structural basis of the conformational and functional regulation of human SERCA2b, the ubiquitous endoplasmic reticulum calcium pump. Bioessays. (2022) 44(7):e2200052. 10.1002/bies.202200052 35560336

[107] Narita T Weinert BT Choudhary C. Author correction: functions and mechanisms of non-histone protein acetylation. Nat Rev Mol Cell Biol. (2019) 20(8):508. 10.1038/s41580-019-0156-9 31267066

[108] Adachi T Weisbrod RM Pimentel DR Ying J Sharov VS Schoneich C S-Glutathiolation by peroxynitrite activates SERCA during arterial relaxation by nitric oxide. Nat Med. (2004) 10(11):1200–7. 10.1038/nm1119 15489859

[109] Tong X Ying J Pimentel DR Trucillo M Adachi T Cohen RA. High glucose oxidizes SERCA cysteine-674 and prevents inhibition by nitric oxide of smooth muscle cell migration. J Mol Cell Cardiol. (2008) 44(2):361–9. 10.1016/j.yjmcc.2007.10.022 18164028 PMC2394666

[110] Paolocci N Katori T Champion HC St John ME Miranda KM Fukuto JM Positive inotropic and lusitropic effects of HNO/NO- in failing hearts: independence from beta-adrenergic signaling. Proc Natl Acad Sci U S A. (2003) 100(9):5537–42. 10.1073/pnas.0937302100 12704230 PMC154380

[111] Tocchetti CG Wang W Froehlich JP Huke S Aon MA Wilson GM Nitroxyl improves cellular heart function by directly enhancing cardiac sarcoplasmic reticulum Ca^2+^ cycling. Circ Res. (2007) 100(1):96–104. 10.1161/01.RES.0000253904.53601.c9 17138943 PMC2769513

[112] Goodman JB Qin F Morgan RJ Chambers JM Croteau D Siwik DA Redox-resistant SERCA [sarco(endo)plasmic Reticulum calcium ATPase] attenuates oxidant-stimulated mitochondrial calcium and apoptosis in cardiac myocytes and pressure overload-induced myocardial failure in mice. Circulation. (2020) 142(25):2459–69. 10.1161/CIRCULATIONAHA.120.048183 33076678 PMC7752816

[113] Sivakumaran V Stanley BA Tocchetti CG Ballin JD Caceres V Zhou L HNO enhances SERCA2a activity and cardiomyocyte function by promoting redox-dependent phospholamban oligomerization. Antioxid Redox Signal. (2013) 19(11):1185–97. 10.1089/ars.2012.5057 23919584 PMC3785857

[114] Kho C Lee A Jeong D Oh JG Chaanine AH Kizana E SUMO1-dependent modulation of SERCA2a in heart failure. Nature. (2011) 477(7366):601–5. 10.1038/nature10407 21900893 PMC3443490

[115] Kim EY Zhang Y Ye B Segura AM Beketaev I Xi Y Involvement of activated SUMO-2 conjugation in cardiomyopathy. Biochim Biophys Acta. (2015) 1852(7):1388–99. 10.1016/j.bbadis.2015.03.013 25857621

[116] Gupta MK Gulick J Liu R Wang X Molkentin JD Robbins J. Sumo E2 enzyme UBC9 is required for efficient protein quality control in cardiomyocytes. Circ Res. (2014) 115(8):721–9. 10.1161/CIRCRESAHA.115.304760 25097219 PMC4180728

[117] Tilemann L Lee A Ishikawa K Aguero J Rapti K Santos-Gallego C SUMO-1 gene transfer improves cardiac function in a large-animal model of heart failure. Sci Transl Med. (2013) 5(211):211ra159. 10.1126/scitranslmed.3006487 24225946

[118] Lee A Jeong D Mitsuyama S Oh JG Liang L Ikeda Y The role of SUMO-1 in cardiac oxidative stress and hypertrophy. Antioxid Redox Signal. (2014) 21(14):1986–2001. 10.1089/ars.2014.5983 24893265 PMC4208582

[119] Oh JG Watanabe S Lee A Gorski PA Lee P Jeong D miR-146a suppresses SUMO1 expression and induces cardiac dysfunction in maladaptive hypertrophy. Circ Res. (2018) 123(6):673–85. 10.1161/CIRCRESAHA.118.312751 30355233 PMC6205728

[120] Kho C Lee A Jeong D Oh JG Gorski PA Fish K Small-molecule activation of SERCA2a SUMOylation for the treatment of heart failure. Nat Commun. (2015) 6:7229. 10.1038/ncomms8229 26068603 PMC4467461

[121] Greenberg B Butler J Felker GM Ponikowski P Voors AA Desai AS Calcium upregulation by percutaneous administration of gene therapy in patients with cardiac disease (CUPID 2): a randomised, multinational, double-blind, placebo-controlled, phase 2b trial. Lancet. (2016) 387(10024):1178–86. 10.1016/S0140-6736(16)00082-9 26803443

[122] Hulot JS Salem JE Redheuil A Collet JP Varnous S Jourdain P Effect of intracoronary administration of AAV1/SERCA2a on ventricular remodelling in patients with advanced systolic heart failure: results from the AGENT-HF randomized phase 2 trial. Eur J Heart Fail. (2017) 19(11):1534–41. 10.1002/ejhf.826 28393439

[123] Lyon AR Babalis D Morley-Smith AC Hedger M Suarez Barrientos A Foldes G Investigation of the safety and feasibility of AAV1/SERCA2a gene transfer in patients with chronic heart failure supported with a left ventricular assist device—the SERCA-LVAD TRIAL. Gene Ther. (2020) 27(12):579–90. 10.1038/s41434-020-0171-7 32669717 PMC7744277

[124] Asokan A Conway JC Phillips JL Li C Hegge J Sinnott R Reengineering a receptor footprint of adeno-associated virus enables selective and systemic gene transfer to muscle. Nat Biotechnol. (2010) 28(1):79–82. 10.1038/nbt.1599 20037580 PMC2912150

[125] Cornea RL Gruber SJ Lockamy EL Muretta JM Jin D Chen J High-throughput FRET assay yields allosteric SERCA activators. J Biomol Screen. (2013) 18(1):97–107. 10.1177/1087057112456878 22923787 PMC3721969

[126] Dahl R. A new target for Parkinson’s disease: small molecule SERCA activator CDN1163 ameliorates dyskinesia in 6-OHDA-lesioned rats. Bioorg Med Chem. (2017) 25(1):53–7. 10.1016/j.bmc.2016.10.008 27776889

[127] Kang S Dahl R Hsieh W Shin A Zsebo KM Buettner C Small molecular allosteric activator of the sarco/endoplasmic Reticulum Ca^2+^-ATPase (SERCA) attenuates diabetes and metabolic disorders. J Biol Chem. (2016) 291(10):5185–98. 10.1074/jbc.M115.705012 26702054 PMC4777852

[128] Nogami K Maruyama Y Sakai-Takemura F Motohashi N Elhussieny A Imamura M Pharmacological activation of SERCA ameliorates dystrophic phenotypes in dystrophin-deficient mdx mice. Hum Mol Genet. (2021) 30(11):1006–19. 10.1093/hmg/ddab100 33822956 PMC8170845

[129] Kaneko M Yamamoto H Sakai H Kamada Y Tanaka T Fujiwara S A pyridone derivative activates SERCA2a by attenuating the inhibitory effect of phospholamban. Eur J Pharmacol. (2017) 814:1–8. 10.1016/j.ejphar.2017.07.035 28734932

[130] Gobbini M Armaroli S Banfi L Benicchio A Carzana G Ferrari P Novel analogues of istaroxime, a potent inhibitor of Na(+), K(+)-ATPase: synthesis, structure-activity relationship and 3D-quantitative structure-activity relationship of derivatives at position 6 on the androstane scaffold. Bioorg Med Chem. (2010) 18(12):4275–99. 10.1016/j.bmc.2010.04.095 20494582

[131] Micheletti R Palazzo F Barassi P Giacalone G Ferrandi M Schiavone A Istaroxime, a stimulator of sarcoplasmic reticulum calcium adenosine triphosphatase isoform 2a activity, as a novel therapeutic approach to heart failure. Am J Cardiol. (2007) 99(2A):24A–32A. 10.1016/j.amjcard.2006.09.003 17239701

[132] Ferrandi M Barassi P Tadini-Buoninsegni F Bartolommei G Molinari I Tripodi MG Istaroxime stimulates SERCA2a and accelerates calcium cycling in heart failure by relieving phospholamban inhibition. Br J Pharmacol. (2013) 169(8):1849–61. 10.1111/bph.12278 23763364 PMC3753840

[133] Carubelli V Zhang Y Metra M Lombardi C Felker GM Filippatos G Treatment with 24 h istaroxime infusion in patients hospitalised for acute heart failure: a randomised, placebo-controlled trial. Eur J Heart Fail. (2020) 22(9):1684–93. 10.1002/ejhf.1743 31975496

[134] Metra M Chioncel O Cotter G Davison B Filippatos G Mebazaa A Safety and efficacy of istaroxime in patients with acute heart failure-related pre-cardiogenic shock—a multicentre, randomized, double-blind, placebo-controlled, parallel group study (SEISMiC). Eur J Heart Fail. (2022) 24(10):1967–77. 10.1002/ejhf.2629 35867804 PMC9804717

[135] Sarma S MacNamara JP Hieda M Howden EJ Lawley JS Livingston S SERCA2a Agonist effects on cardiac performance during exercise in heart failure with preserved ejection fraction. JACC Heart Fail. (2023) 11(7):760–71. 10.1016/j.jchf.2023.02.006 37086245 PMC13524204

[136] Luraghi A Ferrandi M Barassi P Arici M Hsu SC Torre E Highly selective SERCA2a activators: preclinical development of a congeneric group of first-in-class drug leads against heart failure. J Med Chem. (2022) 65(10):7324–33. 10.1021/acs.jmedchem.2c00347 35580334 PMC9150102

[137] Arici M Ferrandi M Barassi P Hsu SC Torre E Luraghi A Istaroxime metabolite PST3093 selectively stimulates SERCA2a and reverses disease-induced changes in cardiac function. J Pharmacol Exp Ther. (2023) 384(1):231–44. 10.1124/jpet.122.001335 36153005

[138] Hamers-Casterman C Atarhouch T Muyldermans S Robinson G Hamers C Songa EB Naturally occurring antibodies devoid of light chains. Nature. (1993) 363(6428):446–8. 10.1038/363446a0 8502296

[139] Scully M Cataland SR Peyvandi F Coppo P Knöbl P Kremer Hovinga JA Caplacizumab treatment for acquired thrombotic thrombocytopenic Purpura. N Engl J Med. (2019) 380(4):335–46. 10.1056/NEJMoa1806311 30625070

[140] De Genst E Foo KS Xiao Y Rohner E de Vries E Sohlmér J Blocking phospholamban with VHH intrabodies enhances contractility and relaxation in heart failure. Nat Commun. (2022) 13(1):3018. 10.1038/s41467-022-29703-9 35641497 PMC9156741

[141] Skogestad J Albert I Hougen K Lothe GB Lunde M Eken OS Disruption of phosphodiesterase 3A binding to SERCA2 increases SERCA2 activity and reduces mortality in mice with chronic heart failure. Circulation. (2023) 147(16):1221–36. 10.1161/CIRCULATIONAHA.121.054168 36876489

[142] Liu Z Bian X Gao W Su J Ma C Xiao X Rg3 promotes the SUMOylation of SERCA2a and corrects cardiac dysfunction in heart failure. Pharmacol Res. (2021) 172:105843. 10.1016/j.phrs.2021.105843 34428586

[143] Wang Z Zeng M Wang Z Qin F Chen J He Z. Dietary luteolin: a narrative review focusing on its pharmacokinetic properties and effects on glycolipid metabolism. J Agric Food Chem. (2021) 69(5):1441–54. 10.1021/acs.jafc.0c08085 33522240

[144] Hu W Xu T Wu P Pan D Chen J Chen J Luteolin improves cardiac dysfunction in heart failure rats by regulating sarcoplasmic reticulum Ca(2+)-ATPase 2a. Sci Rep. (2017) 7:41017. 10.1038/srep41017 28112209 PMC5253630

[145] Du Y Liu P Xu T Pan D Zhu H Zhai N Luteolin modulates SERCA2a leading to attenuation of myocardial ischemia/reperfusion injury via sumoylation at lysine 585 in mice. Cell Physiol Biochem. (2018) 45(3):883–98. 10.1159/000487283 29421780

[146] Lamberti MJ Morales Vasconsuelo AB Chiaramello M Ferreira VF Macedo Oliveira M Baptista Ferreira S NQO1 induction mediated by photodynamic therapy synergizes with beta-lapachone-halogenated derivative against melanoma. Biomed Pharmacother. (2018) 108:1553–64. 10.1016/j.biopha.2018.09.159 30372857

[147] Sulaiman M Matta MJ Sunderesan NR Gupta MP Periasamy M Gupta M. Resveratrol, an activator of SIRT1, upregulates sarcoplasmic calcium ATPase and improves cardiac function in diabetic cardiomyopathy. Am J Physiol Heart Circ Physiol. (2010) 298(3):H833–43. 10.1152/ajpheart.00418.2009 20008278 PMC2838561

[148] Dong Q Wu Z Li X Yan J Zhao L Yang C Resveratrol ameliorates cardiac dysfunction induced by pressure overload in rats via structural protection and modulation of Ca(2+) cycling proteins. J Transl Med. (2014) 12:323. 10.1186/s12967-014-0323-x 25425099 PMC4278670

[149] Gal R Deres L Horvath O Eros K Sandor B Urban P Resveratrol improves heart function by moderating inflammatory processes in patients with systolic heart failure. Antioxidants (Basel. (2020) 9(11):1108. 10.3390/antiox9111108 33187089 PMC7696241

[150] O'Donnell JM Fields A Xu X Chowdhury SA Geenen DL Bi J. Limited functional and metabolic improvements in hypertrophic and healthy rat heart overexpressing the skeletal muscle isoform of SERCA1 by adenoviral gene transfer in vivo. Am J Physiol Heart Circ Physiol. (2008) 295(6):H2483–94. 10.1152/ajpheart.01023.2008 18952713 PMC2614552

[151] Teucher N Prestle J Seidler T Currie S Elliott EB Reynolds DF Excessive sarcoplasmic/endoplasmic reticulum Ca^2+^-ATPase expression causes increased sarcoplasmic reticulum Ca^2+^ uptake but decreases myocyte shortening. Circulation. (2004) 110(23):3553–9. 10.1161/01.CIR.0000145161.48545.B3 15505097

[152] Carvalho BM Bassani RA Franchini KG Bassani JW. Enhanced calcium mobilization in rat ventricular myocytes during the onset of pressure overload-induced hypertrophy. Am J Physiol Heart Circ Physiol. (2006) 291(4):H1803–13. 10.1152/ajpheart.01345.2005 16648178

